# A female case report of LGMD2B with compound heterozygous mutations of the *DYSF* gene and asymptomatic mutation of the X-linked *DMD* gene

**DOI:** 10.3389/fneur.2023.1213090

**Published:** 2023-09-27

**Authors:** Xiaojie Cao, Li Zeng, Zhijie Lu, Jin Fan, Song Tan, Mingjie Zhang, Zegang Yin

**Affiliations:** ^1^Department of Neurology, The General Hospital of Western Theater Command, Chengdu, Sichuan Province, China; ^2^Department of Neurology, Sichuan Academy of Medical Science and Sichuan Provincial People's Hospital, University of Electronic Science and Technology of China, Chengdu, Sichuan Province, China

**Keywords:** LGMD, DMD/BMD, dysferlin, dysferlinopathy, *DYSF* gene, case report

## Abstract

We report the case of a 31-year-old Chinese woman with a chief complaint of weakness in the lower limbs, which was diagnosed as limb-girdle muscular dystrophy 2B (LGMD2B) with compound heterozygous mutations of the *DYSF* gene. Meanwhile, this woman is an asymptomatic carrier with the mutation of the X-linked *DMD* gene. The electromyography, muscle MRI, and muscle biopsy indicated a chronic myogenic injury with dysferlin deletion. As a result of genetic testing, compound heterozygous G-to-T base substitution at position 5,497 in exon 49 of the *DYSF* gene, leading to a codon change from glutamic acid to termination codon at position 1,833, and a heterozygous C-to-G base change at position 4,638 + 8 in intron 42 of the *DYSF* gene with a consequence of splice, which has never been reported, were identified as candidate causative mutations. Unfortunately, *DMD* gene mutation c.3921+12A>G of the *DMD* gene on the X chromosome was also found in this patient. Finally, the patient was diagnosed as LGMD2B clinically and genetically. In the previous 2 years, the patient's lower limb weakness became slightly worse, resulting in even the total distance walked than before. Fortunately, during the follow-up, her son had not shown slowness or limitation of movement. Genetic testing by next-generation sequencing confirmed the final diagnosis of LGMD2B, and we identified the novel compound heterozygous variants in the *DYSF* gene, which is of great significance to the accurate diagnosis of genetically coded diseases. Much attention needs to be paid in clinics toward hereditary neuromuscular diseases with multiple pathogenic gene mutations. Genetic counseling and clinical follow-up should be the priorities in future, and promising treatments are also worth exploring.

## Introduction

Limb-girdle muscular dystrophy (LGMD) is a group of rare, genetically determined degenerative muscle disorders with high genetic heterogeneity and phenotypic diversity. The main clinical manifestations are weakness and atrophy of predominant pelvic and shoulder muscles, with the typical symptom of difficulty climbing stairs. Among 11 studies included in a meta-analysis including all age groups in 2016, the pooled prevalence of LGMD was 1.63 per 100,000 (1). According to a genetic pattern, LGMD can be divided into the autosomal dominant inheritance (LGMD1) and autosomal recessive inheritance (LGMD2) forms. The LGMD2 form currently accounts for 90% of total LGMD patients and has an earlier onset and faster progression (2). LGMD2B and LGMD2A accounted for 75% of the total LGMDs, as the most common LGMD subtypes in a previous China-based cohort (3). LGMD2B, the more frequently diagnosed subtype of the two, is caused by homozygous or complex heterozygous mutations of the *Dysferlin* gene (*DYSF*), resulting in dysferlin protein deficiency or dysfunction (4).

Duchenne muscular dystrophy (DMD) is a rare X-linked recessive muscle degenerative disorder found predominantly in male children. The frequent clinical manifestations of DMD are proximal muscle weakness and wasting, gastrocnemius muscle hypertrophy, and gait abnormality, seriously affecting daily motor abilities. DMD is caused by mutations of the *Dystrophin* gene (*DMD*) in the Xp21.2 region that cause the absence of dystrophin protein or structural defects of the cytoskeleton (5). As reported in an updated meta-analysis, the pooled global DMD prevalence was 7.1 per 100,000 men, while the birth prevalence was 19.8 per 100,000 live male births (6). Usually, patients with DMD lose independent ambulation within the age of 12 or 13 years and die mainly due to cardiorespiratory failure before the age of 30 years. In comparison, Becker muscular dystrophy (BMD) is the allelic form characterized by a more benign clinical course (5).

LGMD and DMD/BMD are different types of progressive muscular dystrophies in consideration of age at onset, atrophic muscle distribution, disease progression, and prognosis. In this current report, we describe the case of a woman with a chief complaint of weakness in her lower limbs, which was finally diagnosed as LGMD2B with compound heterozygous mutations of the *DYSF* gene in combination with other means of inspection. Furthermore, this woman is an asymptomatic carrier with a mutation of the X-linked *DMD* gene, which is relatively uncommon in clinical practice. This report focuses on the diagnostic process of the female case and her family and attracts increasing attention to hereditary neuromuscular diseases with multiple pathogenic gene mutations.

## Case presentation

A 31-year-old Chinese woman was admitted to the Department of Neurology of our hospital on 2 April 2021, with a chief complaint of lower limb weakness. More than 1 year before admission, the patient found that she had to pull the handrail to get on the bus because of the weakness of her lower limbs. Before 1 year, she began suffering from low back pain and experiencing difficulty in standing after squatting and weakness in her lower limbs when climbing uphill or stairs. The abovementioned symptoms were getting worse in the past 6 months. Unfortunately, she was unable to stand up after squatting, and she had to bend over and put her hands on her thighs when climbing uphill or stairs at the time of admission. The patient was gradually unable to perform coordinated running and jumping movements. There was no obvious abnormality when walking on a flat road. This patient was previously healthy. Her parents, her younger brother, her daughter, and her son had no similar symptoms.

On the day of admission, the patient's vital signs were normal. The neurological examination of the cranial nerves was negative. The patient had difficulty climbing the stairs or standing up after squatting during the physical examination. Movements of the hips and knees were slightly limited, while the feet were not. The proximal muscle strength of both lower limbs (mainly including the pelvic girdle and quadriceps muscles) was level 4 (0–5), and the distal muscle strength was level 5. The muscle strength of both upper limbs was normal. There were no sensory deficits in the four limbs. The tendon reflexes of both lower limbs were weakened, and the upper limbs were normal. The bilateral Babinski signs were negative. Gowers' sign was positive.

Laboratory data showed increased levels of glutamic-pyruvic transaminase (126.3 IU/L), glutamic-oxalacetic transaminase (122.8 IU/L), creatine kinase (CK, 8950.4 IU/L), myohemoglobin (846.140 ug/L), creatine kinase isoenzyme (94.960 ug/L) but decreased counts of white blood cell (3.62^*^10^∧^9/L), red blood cell (3.95^*^10^∧^12/L), hemoglobin concentration (97 g/L), red blood cell-specific volume (31.2%), neutrophil (1.74^*^10^∧^9/L), mean red blood cell volume (78.9 fL), mean red blood cell hemoglobin content (24.6 pg), and mean corpuscular-hemoglobin concentration (312 g/L). Blood routine examination indicated iron deficiency anemia, thus ferritin (3.40 ng/ml), serum iron (4.58 umol/L), iron saturation (7.5%), unsaturated iron (56.79 umol/L), and total iron binding (61.37 umol/L) were further measured. In addition, autoantibody test results showed that the anti-nuclear antibody (1:100) and anti-mitochondrial antibody (M2) were positive. Anti-Ha/Tyr antibody IgG was also positive in the idiopathic inflammatory myopathy (IIM) spectrum test from the serum. Comprehensive infectious, paraneoplastic, and inflammatory examinations of the patient were negative.

**Figure 1:**
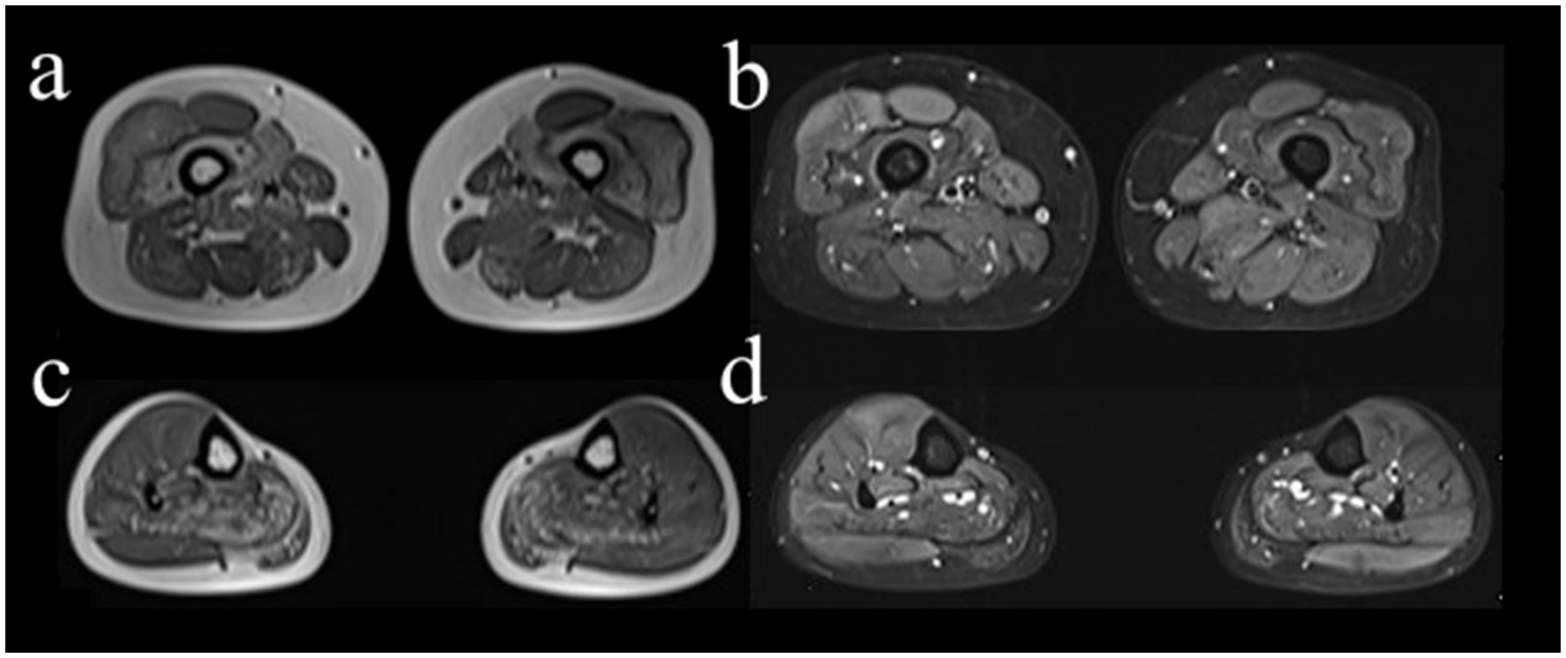
Axial MRI images of right lower limb muscles. The right vastus intermedius muscle **(a, b)**, medial head of gastrocnemius muscle, and soleus muscle **(c, d)** showed spotted fatty degeneration. The MRI sequences were T1-weighted imaging **(a, c)** and fat-suppressed T2-weighted imaging **(b, d)**, respectively.

Electrophysiological tests showed that the motor and sensory nerves of the limbs were normal. On needle electromyography (EMG), the spontaneous potentials of the muscles were not observed in the resting state. During light contraction, the mean amplitudes of the bilateral gastrocnemius and iliopsoas muscles and the left gluteus maximus decreased, and the mean time limit was narrow. These results indicated myogenic injury. There were no abnormal signs on cranial, cervical, and lumbar spine magnetic resonance imaging (MRI). To confirm the type of myopathy, we selectively performed the right limb muscle MRI, which showed that the muscle groups (anterior and posterior thigh and also posterior calf) were amyotrophic and spotted fatty degeneration, accompanied by partial muscle edema (Figure 1). Meanwhile, a muscle MRI of the right upper arm showed slight edema.

**Figure 2:**
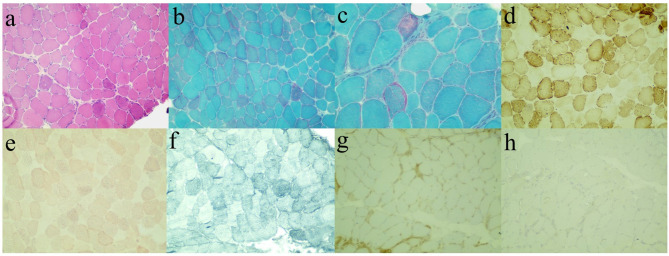
Immunohistochemical staining images of left gastrocnemius muscle biopsy. Hematoxylin-eosin (HE, **a**), modified Gomori trichrome (MGT, **b, c**), cytochrome C oxidase (COX, **d**), oil red O (ORO, **e**), Sudan black B (SBB, **f**), major histocompatibility complex class I (MHC I, **g**), and dysferlin **(h)**.

While combing through the current information analysis, we suspected that the patient suffered from muscular dystrophy. To confirm the hypothesis, muscle biopsy (Figure 2) and genetic testing were furtherly performed after getting approval. Hematoxylin-eosin (HE)-stained images of left gastrocnemius muscle biopsy showed muscle cells of slightly variable size and irregular shape and scattered necrotic fibers without the increased endomysial connective tissue and lymphocytic infiltration. Modified Gomori trichrome (MGT)-stained images showed sporadic ragged-red fibers (RRFs), while no small rod-like particles or rimmed vacuoles were observed. In addition, no evident abnormality was shown in the other histochemical staining techniques involving a reduced form of nicotinamide adenine dinucleotide (NADH), cytochrome C oxidase (COX), succinate dehydrogenase (SDH), periodic acid Schiff (PAS), oil red O (ORO), Sudan black B (SBB), and ATPase. Finally, immunohistochemical data demonstrated the absence of major histocompatibility complex class I (MHC I), myxovirus resistance protein A (MxA), membrane attack complex (MAC), and dysferlin. These results were consistent with the chronic myogenic injury with dysferlin deletion. In consideration of RRFs observed usually in mitochondrial damage, an additional complementary blood test was conducted. However, none of the mutations in the mitochondrial genome was detected in the genetic screening of the pathogenic mutations in the MITOMAP database.

**Figure 3:**
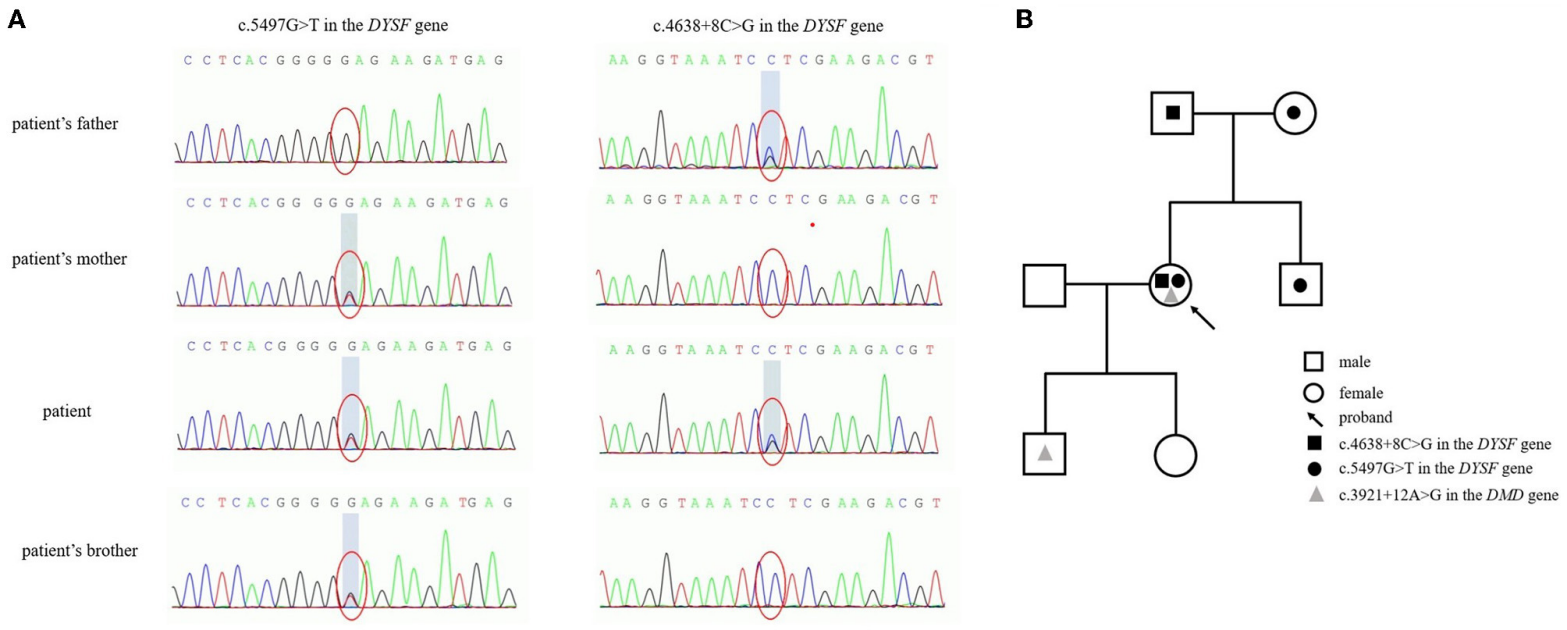
Genetic mutations in the *DYSF* gene **(A)** and a pedigree chart **(B)** to illustrate the genetic inheritance among the patient and her family members.

As a genetic testing result (Figure 3A), compound heterozygous G-to-T base substitution at position 5,497 in exon 49 of the *DYSF* gene, leading to a codon change from glutamic acid to termination codon at position 1,833 (NM_003494.3: c.5497G>T, p.Glu1833Ter), and a heterozygous C-to-G base change at position 4,638 + 8 in intron 42 of the *DYSF* gene with a consequence of splice, which has never been reported, were identified as candidate causative mutations. According to related guides of the American College of Medical Genetics and Genomics (ACMG), the former mutation is pathogenic and the latter mutation is a variation of unknown clinical significance (VUS). The variant naming rules refer to Human Genome Variation Society (HGVS) recommendations. Unfortunately, *DMD* gene mutation c.3921+12A>G of the *DMD* gene on the X chromosome was also found in this patient. Further genealogical verification confirmed that c.5497G>T in the *DYSF* gene was derived from her mother and c.4638+8C>G was derived from her father, while there was no mutation in the *DMD* gene of her parents. Only c.5497G>T in the *DYSF* gene was identified in her younger brother without muscular atrophy symptoms. To further investigate the possibility of muscular dystrophy in the offspring, the son of the patient completed genetic verification and carried the same *DMD* gene mutation on the X chromosome. A pedigree chart was shown in Figure 3B to illustrate the genetic inheritance among the patient and her family members.

Finally, the patient was diagnosed as LGMD2B clinically and genetically. We offered symptomatic treatment during hospitalization, but the muscle weakness did not improve remarkably. We recommended regular medical follow-up for this patient after discharge. To date, the patient's lower limb weakness has become slightly worse, resulting in the walking distance becoming even shorter than before.

## Discussion

Progressive muscular dystrophies comprise a group of inherited and degenerative diseases characterized by progressive muscle weakness and atrophy that induces dyskinesia. According to age at onset, atrophic muscle distribution, disease progression, and prognosis, progressive muscular dystrophies include DMD/BMD, LGMD, facioscapulohumeral muscular dystrophy (FSHD), Emery–Dreifuss muscular dystrophy (EDMD), congenital muscular dystrophy (CMD), oculopharyngeal muscular dystrophy (OPMD), distal muscular dystrophy, and other rare types (7–11).

LGMD mainly manifests as weakness and atrophy of predominantly pelvic and shoulder muscles with different genetic patterns and gene mutations. The current diagnosis of LGMD is based on clinical manifestations and genetic inheritance, besides laboratory criteria, such as elevated serum CK, muscle biopsy, histopathological features, and muscle imaging changes (2). The female patient discussed above suffered from proximal muscle weakness of both lower limbs with insidious onset, which aggravated gradually from the age of 30 years of the patient. Laboratory data showed elevated serum CK detected in the blood. In addition, EMG results indicated that muscular damage and right limb MRI showed widely spread muscular dystrophy. Furthermore, muscle biopsy indicated chronic myogenic injury with dysferlin deletion in accordance with dystrophic changes. This patient was therefore diagnosed as suffering from dysferlinopathy.

Dysferlinopathies are a group of autosomal recessive disorders characterized by the involvement of proximal and/or distal limb-girdle muscles caused by dysferlin deficiency/mutations (2, 4). Dysferlinopathy phenotypes at onset include LGMD2B, distal Miyoshi Myopathy (MM), proximal-distal limb-girdle phenotype, distal anterior compartment myopathy (DACM), and other less frequent phenotypes. In general, patients with LGMD2B present with weakness that is first evident in the proximal muscles of the pelvic girdle, leading to difficulties in walking upstairs or running (12). The age of LGMD2B onset is in the late teens or early adulthood. Moreover, the shoulder girdle and upper limb muscles are frequently involved later, while facial, neck, and hand muscles are usually unaffected during the course of the disease (4). Based on the clinical characteristics and the results mentioned above, the patient was diagnosed with LGMD2B.

Before the muscle biopsy and genetic testing, however, polymyositis (PM) should be considered in the differential diagnosis. PM is a CD8 (+) T cell-mediated autoimmune disease clinically manifested by symmetrical and proximal muscle weakness (13). LGMD2B and PM have some common clinical features, including weakness of proximal limbs, increased CK level, EMG changes indicating muscle-derived injury, and MRI changes in the affected muscles (14). Furthermore, the positive myositis-specific autoantibody such as anti-Ha/Tyr antibody makes PM highly suspicious. However, there were still many points of disapproval. This patient did not suffer from neck flexor weakness, muscle tenderness, and other systemic symptoms along with elevated inflammatory or infectious indicators. Finally, the critical methods for distinguishing these two diseases are muscle biopsy and genetic testing of dysferlin deficiency/mutations.

Dysferlin, a protein encoded by the skeletal muscle gene *DYSF* in the 2p13 region, is mutated in dysferlinopathies (15). It has been identified as a crucial member of membrane repair, vesicle trafficking, and membrane remodeling in the skeletal muscle (16, 17). Moreover, dysferlin deficiency causes the activation of the muscle inflammasome to generate a proinflammatory environment and exacerbate muscle damage (18). As expected, immunohistochemical staining images of the left gastrocnemius muscle biopsy showed chronic myogenic injury with dysferlin deletion and no lymphocytic infiltration or MHC I expression, which could complement the evidence to discard the PM diagnosis.

A growing number of studies are being devoted to mitochondrial diseases (MIDs), especially those involving the neuromuscular system (19). MIDs are a group of genetically defective disorders that result from structural and functional alterations of the mitochondria, which then lead to respiratory chain and energy metabolism dysfunctions. In addition to molecular genetic tests as the gold standard, pathological examinations following a muscle biopsy can complement the diagnosis of mitochondrial myopathies, including (1) RRFs, (2) mitochondria proliferation observed in SDH staining often described as ragged-blue fibers, (3) COX deficient or negative, and (4) ultrastructurally abnormal mitochondria often with paracrystalline inclusions (20). The extent of mitochondrial abnormalities in dysferlinopathy is presently unclear but attracts sufficient interest (21). A previous LGMD case report has shown a mitochondrial deficit in histomorphology characterized by the presence of RRFs, deficiency of COX, and abnormal ultrastructure of mitochondria (22). Moreover, RRFs in the skeletal muscle of dysferlinopathy have been reported (23). Similarly, more cases with dysferlinopathy reported in a Chinese-based study displayed a higher proportion of RRFs and COX-deficient fibers in muscle pathologies (24). In consideration of sporadic RRFs observed in MGT-stained images of the present patient, additional gene sequencing was conducted, and no mitochondrial DNA mutations were found. Primary mitochondrial myopathy was not supported based on the molecular genetic test, but secondary muscle mitochondrial dysfunction could not be completely excluded.

In the last decades, Asian and European countries have conducted nationwide multicenter genetic studies regarding the mutations of the *DYSF* gene involving dozens to hundreds of patients. In world patients with dysferlinopathy, the exons with the most *DYSF* gene variants were exon 29, exon 39, exon 45, and exon 6, and the most common consequences were missense, splice, and frameshift variants (25). Reported in a Japanese cohort, the c.2997G > T (p.Trp999Cys), c.1566C > G (p.Tyr522Ter), and c.3373del (p.Glu1125Lys) variants were common in the MM subtype, while the c.2997G > T variant was definitely the most common in the LGMD2B subtype (26). However, c.1375dup was identified as the hotspot in the Chinese dysferlinopathy cohort, differing from the hotspot c.2997G>T in world patients (25). Hence, we reported the compound heterozygous mutations, c.5497G>T and c.4638+8C>G, in the *DYSF* gene of this patient. Additionally, c.5497G>T was a known nonsense mutation (15), while c.4638+8C>G was a splice mutation that has not been reported in any public databases. The parents and a brother of this patient were screened for further genealogical verification. The results showed that the mutations of this patient were derived from her parents, and her younger brother was a pathogenic mutation carrier without muscular atrophy symptoms, which confirmed that this disease accorded with the autosomal recessive inheritance rule. Therefore, gene sequencing confirmed her final diagnosis of LGMD2B and identified the novel compound heterozygous variants in the *DYSF* gene.

According to the autosomal recessive inheritance rules, the offspring of the patient will not suffer from the disease if her husband is not a pathogenic gene mutation carrier. However, unfortunately, the patient carried a *DMD* gene mutation c.3921+12A>G on the X chromosome, which has been reported earlier (27). It is reasonable to presume that this mutation can only come from her mother. Variants in the *DMD* gene caused X-linked recessive DMD/BMD characterized by progressive muscle degeneration and weakness. DMD/BMD generally affects men only, but there are still some female carriers experiencing muscle weakness (28). As a heterozygous carrier, this patient passed the genetic disease on to her son with a 50% chance, and her daughter has a 50% chance of being a carrier. To conduct appropriate genetic counseling, further genealogical verification was conducted and identified that the son of the patient has carried the same *DMD* gene mutation regrettably. To date, her 5-year-old son has not shown slowness or limitation of movement.

Hereditary neuromuscular disorders are a wide-ranging group of diseases influencing the life quality of patients severely. With the development of detection technology, hundreds of related genes and many more pathogenic variants are discovered. Genetic testing by the next-generation sequencing method to identify the pathogenic mutations is of great significance to the accurate diagnosis of genetically coded diseases. LGMD2B and DMD are different types of progressive muscular dystrophies and are caused by mutations to the *dysferlin* and *dystrophin* genes, respectively. In this current report, we reported a Chinese woman who was finally diagnosed as LGMD2B with the compound heterozygous mutations, c.5497G>T and c.4638+8C>G, in the *DYSF* gene. Additionally, c.4638+8C>G is a novel mutation and broadens the genetic spectrum of LGMD2B. Furthermore, this woman is an asymptomatic carrier with a mutation of the X-linked *DMD* gene and has passed the pathogenic mutation on to her son. A case of neuromuscular disease suffering multiple genetic pathogenic risks is relatively uncommon in clinical practice. Much attention must be paid in clinics toward hereditary neuromuscular diseases with multiple pathogenic gene mutations. Genetic counseling and clinical follow-up are the priorities in future, and promising treatments are also worth exploring.

## Data availability statement

The raw sequence data reported in this paper have been deposited in the Genome Sequence Archive (Genomics, Proteomics & Bioinformatics 2021) in National Genomics Data Center (Nucleic Acids Res 2021), China National Center for Bioinformation/Beijing Institute of Genomics, Chinese Academy of Sciences (GSA: HRA005262) that are controlled accessible at (NGDC: https://ngdc.cncb.ac.cn/). Meanwhile, the raw sequence data can also be accessed after an approval application to the China National GeneBank DataBase (CNGBdb, https://db.cngb.org/). Please refer to https://db.cngb.org/, or email: CNGBdb@cngb.org for detailed application guidance. The accession number CNP0004583 should be included in the application.

## Ethics statement

The studies involving human participants were reviewed and approved by the General Hospital of Western Theater Command. The patients/participants provided their written informed consent to participate in this study. Written informed consent was obtained from the individual(s) for the publication of any potentially identifiable images or data included in this article.

## Author contributions

XC, ZL, and JF were responsible for the patient's management during hospitalization. LZ and ST performed muscle biopsy and further interpreted the results. XC and MZ prepared the manuscript. XC, LZ, MZ, and ZY reviewed and made significant contributions to the final version. All authors approved the final version of the manuscript.
